# Comment on “Diagnosis, prevalence, and mortality of sarcopenia in dialysis patients: a systematic review and meta‐analysis” by Shu et al.

**DOI:** 10.1002/jcsm.13113

**Published:** 2023-09-27

**Authors:** Fan Zhang, Huachun Zhang

**Affiliations:** ^1^ Department of Nephrology Longhua Hospital Shanghai University of Traditional Chinese Medicine Shanghai China; ^2^ Department of Nursing Longhua Hospital Shanghai University of Traditional Chinese Medicine Shanghai China

Dear editor:

We read the article with great interest published in *Journal of Cachexia Sarcopenia and Muscle* by Shu et al.^1^ This systematic review and meta‐analysis demonstrated that the prevalence of sarcopenia in dialysis patients was approximately 28.5% (95% CI 22.9–34.1%) and was not associated with mean age (regression coefficient 0.004; 95% CI: −0.005–0.012) and dialysis duration (regression coefficient 0.002; 95% CI −0.002–0.005). In addition, sarcopenia was strongly associated with high mortality risk in dialysis patients. Nevertheless, we thought there was something to discuss.

First, the authors searched the literature without language restriction, but only Embase, MEDLINE, PubMed and Cochrane Library were searched. Obtaining grey literature by searching clinical trial registries (e.g. ClinicalTrials.gov) is also a necessary step for systematic review.^2^

Second, we have doubts about *Figure S3* in the original paper. The authors plotted different subgroup forests in the same figure, and there should not be an additional overall, which is a misconception. And what should be the interpretation of the resulting aggregated prevalence (0.30; 95% CI 0.26, 0.34)?

Third, studies have shown that the prevalence of sarcopenia is higher in female dialysis patients.^3^, ^4^ We note that the meta‐analysis by Shu et al.^1^ included a relatively high proportion of males in studies ranging from 44.0% to 100%, and the authors' results may have underestimated the actual prevalence. The authors should have discussed this.

## Conflict of interest

None declared.

## Funding

This study was supported by grants from Longhua Hospital Shanghai University of Traditional Chinese Medicine (Y21026).

## References

[1] Shu X , Lin T , Wang H , Zhao Y , Jiang T , Peng X , et al. Diagnosis, prevalence, and mortality of sarcopenia in dialysis patients: A systematic review and meta‐analysis. J Cachexia Sarcopenia Muscle 2022;13:145–158. 34989172 10.1002/jcsm.12890 PMC8818609

[2] Lefebvre C , Glanville J , Briscoe S , Featherstone R , Littlewood A , Marshall C , et al. Searching for and selecting studies. In Higgins JPT , Thomas J , Chandler J , Cumpston M , Li T , Page MJ , Welch VA , eds. Cochrane handbook for systematic reviews of interventions version 6.3 (updated February 2022). Cochrane; 2022. Available from: www.training.cochrane.org/handbook

[3] Umakanthan M , Li JW , Sud K , Duque G , Guilfoyle D , Cho K , et al. Prevalence and factors associated with sarcopenia in patients on maintenance dialysis in Australia‐A single centre, cross‐sectional study. Nutrients 2021;13:3284. 34579163 10.3390/nu13093284 PMC8469859

[4] Kurita N , Wakita T , Fujimoto S , Yanagi M , Koitabashi K , Suzuki T , et al. Hopelessness and depression predict sarcopenia in advanced CKD and dialysis: A multicenter cohort study. J Nutr Health Aging 2021;25:593–599.33949624 10.1007/s12603-020-1556-4

